# Risk Profiles for Endometriosis in Japanese Women: Results From a Repeated Survey of Self-Reports

**DOI:** 10.2188/jea.JE20140124

**Published:** 2015-03-05

**Authors:** Toshiyuki Yasui, Kunihiko Hayashi, Kazue Nagai, Hideki Mizunuma, Toshiro Kubota, Jung-Su Lee, Shosuke Suzuki

**Affiliations:** 1 Department of Reproductive Technology, Institute of Health Biosciences, The University of Tokushima Graduate School, Tokushima, Japan; 1 徳島大学大学院ヘルスバイオサイエンス研究部生殖補助医療学分野; 2 Department of Basic Medical Sciences, School of Health Sciences, Gunma University, Maebashi, Japan; 2 群馬大学大学院保健学研究科; 3 Department of Obstetrics and Gynecology, Hirosaki University School of Medicine, Hirosaki, Aomori, Japan; 3 弘前大学医学部産科婦人科; 4 Department of Comprehensive Reproductive Medicine, Tokyo Medical and Dental University, Tokyo, Japan; 4 東京医科歯科大学大学院医歯学総合研究科生殖機能協関学; 5 Department of Health Promotion Science, School of Public Health, University of Tokyo, Tokyo, Japan; 5 東京大学大学院医学系研究科公共健康医学専攻健康増進科学分野; 6 Faculty of Medicine, Gunma University, Maebashi, Japan; 6 群馬大学名誉教授

**Keywords:** endometriosis, risk factors, validation

## Abstract

**Background:**

The prevalence and risk factors for endometriosis may differ according to diagnosis methodologies, such as study populations and diagnostic accuracy. We examined risk profiles in imaging-diagnosed endometriosis with and without surgical confirmation in a large population of Japanese women, as well as the differences in risk profiles of endometriosis based on history of infertility.

**Methods:**

Questionnaires that included items on sites of endometriosis determined by imaging techniques and surgical procedure were mailed to 1025 women who self-reported endometriosis in a baseline survey of the Japan Nurses’ Health Study (*n* = 15 019).

**Results:**

Two hundred and ten women had surgically confirmed endometriosis (Group A), 120 had imaging-diagnosed endometriosis without a surgical procedure (Group B), and 264 had adenomyosis (Group C). A short menstrual cycle at 18–22 years of age and cigarette smoking at 30 years of age were associated with significantly increased risk of endometriosis (Group A plus Group B), while older age was associated with risk of adenomyosis (Group C). In women with a history of infertility, a short menstrual cycle was associated with a significantly increased risk of endometriosis in both Group A and Group B, but risk profiles of endometriosis were different between Group A and Group B in women without a history of infertility.

**Conclusions:**

Women with surgically confirmed endometriosis and those with imaging-diagnosed endometriosis without surgery have basically common risk profiles, but these risk profiles are different from those with adenomyosis. The presence of a history of infertility should be taken into consideration for evaluation of risk profiles.

## Abstract (ja)

**背景:**

子宮内膜症の発症や危険因子に関する研究結果は、対象者の選択や子宮内膜症の診断方法によって異なる可能性が考えられる。私たちは本邦女性において画像検査によって診断された子宮内膜症と手術によって診断された子宮内膜症について、リスク因子が異なるのかどうかを検討し、同時に子宮内膜症に影響を及ぼす不妊症の既往の有無によってそれらのリスク因子に違いが存在するかどうかを検討した。

**方法:**

日本ナースヘルス研究のベースライン調査（14945名）において子宮内膜症と回答した1025名に対して、画像検査や手術に関する内容や部位を含めた子宮内膜症に関する詳細な質問票を送り、回答してもらった。

**結果：:**

手術により子宮内膜症と診断された群（A群）は210名、手術は行わず画像検査のみで子宮内膜症と診断された群（B群）は120名、子宮腺筋症と診断された群（C群）は264名であった。子宮内膜症と診断されたA群＋B群におけるリスク因子は、18～22歳時における短い月経周期、30歳時における喫煙習慣であったが、子宮腺筋症であるC群では年齢がリスク因子となっており、両疾患におけるリスク因子は異なっていた。また、不妊症の既往がある女性では、A群およびB群いずれも、18～22歳時における短い月経周期がリスク因子であったが、不妊症の既往がない女性ではA群およびB群とでリスク因子が異なっていた。

**結論:**

手術によって子宮内膜症と診断された群と画像検査のみで子宮内膜症と診断された群はいずれも基本的には共通のリスク因子を有しており、子宮腺筋症のリスク因子とは異なっていた。また、子宮内膜症のリスク因子を検討するためには不妊症の既往の有無を考慮すべきである。

## INTRODUCTION

Endometriosis is defined as the presence of endometrial-like tissue outside the uterine cavity and is associated with symptoms of dysmenorrhea, dyspareunia, chronic pain, and infertility. The estimated prevalence of endometriosis varies by population. Among women with pelvic pain, the prevalence of endometriosis ranged from about 5% to 21%.^1^^,^^2^ The prevalence of pelvic endometriosis was 6%–10% in women of reproductive age.^3^ Missmer et al reported that the prevalence of physician-diagnosed endometriosis was about 5%.^4^ Eskenazi et al reported that the estimated prevalence of endometriosis in the general population was 10% based on a single cohort study.^5^ Recently, the incidence of magnetic resonance imaging (MRI)-diagnosed endometriosis was reported to be 11% in a population cohort.^6^

Results regarding risk factors of endometriosis have also been controversial. Since previous epidemiological studies on the prevalence and risk factors of endometriosis have been based on clinically diagnosed endometriosis, self-reported physician-diagnosed endometriosis without confirmation by a surgical procedure may be substantially misclassified. It has been reported that the selection of appropriate controls has been a problem in case-control studies and that controls should be selected from the source population from which cases were selected.^7^ In prospective studies carried out to clarify the associations of endometriosis with endometriosis-related diseases, the subject population consisted of subjects with surgically confirmed endometriosis but non-surgically confirmed subjects. An invasive procedure and histological examination are needed for a definitive diagnosis of endometriosis but are not performed for women with minimal or mild endometriosis who have no symptoms. Prospective studies including women with early-stage endometriosis cannot easily be performed, since this type of invasive case finding is not performed in asymptomatic patients and patients with severe endometriosis are likely to have received a surgical procedure.^8^ Studies are unable to assess risks of occurrence of endometriosis-related diseases when the endometriosis has been treated by a surgical procedure. Therefore, a validated method of diagnosing endometriosis using non-invasive imaging is needed. Eskenazi et al reported that noninvasive procedures such as history-taking, pain report, physical examination, and ultrasound sonography have had moderate success in predicting surgical diagnosis of ovarian endometriosis.^9^ It has been reported that the sensitivity and specificity of MRI relative to histologically confirmed endometriosis were 69% and 75%, respectively, and that the sensitivity of MRI was 76.9% for endometriosis detected by laparoscopy.^10^^,^^11^

On the other hand, endometriosis causes infertility with dysmenorrhea, pelvic pain, and dyspareuria in women of reproductive age. Approximately 20% of infertile women have been found to have endometriosis.^12^ Recently, Peterson et al reported that a history of infertility was a consistent risk factor for endometriosis in both an operative cohort and a population cohort who underwent MRI.^13^ Women without a history of infertility determined by laparoscopy may be symptomatic, whereas those with a history of infertility may be asymptomatic, suggesting that risk factors for endometriosis with infertility differ from those for endometriosis without infertility. Missmer et al reported that the risk of endometriosis among women with red hair as a natural hair color differed by infertility status.^14^ Therefore, infertility status should be considered in epidemiological studies on endometriosis.

To clarify the differences in prevalence and risk factors for endometriosis between surgically confirmed endometriosis and imaging-diagnosed endometriosis, we compared risk profiles in imaging-diagnosed endometriosis with and without a surgical procedure in a large population of Japanese women. We also examined the differences in risk profiles of endometriosis according to the presence of a history of infertility.

## METHODS

### Data collection

The Japan Nurses’ Health Study (JNHS) is a large prospective cohort study designed to investigate the effects of lifestyle and healthcare practices on the health of Japanese women.^15^ A baseline survey was conducted from 2001 to 2007, and a 10-year follow-up is ongoing. The study population was comprised of female registered nurses, licensed practical nurses, public health nurses, and/or midwives who were at least 25 years of age and residents in any of the 47 prefectures in Japan at the baseline survey. The JNHS coordination and data center is located at the Epidemiological Research Office, School of Health Sciences, Gunma University.

Personal information, occupation, and information on physical indicators as well as results of periodic medical examinations, habits and lifestyle, history of reproductive health, use of female hormone agents, use of other drugs and supplements, and medical history and family history of diseases was collected through self-administered questionnaires at the time of cohort enrollment. Basic information on medical, anthropometric, reproductive, and dietary factors, including body weight and height at 18 years of age, history of oral contraceptive use, cigarette smoking, parity, and age at menarche, was also collected. With regard to regularity of the menstruation cycle, we asked about cycle regularity at 18–22 years of age. Body mass index (BMI) was calculated as weight (kg)/height (m)^2^ using self-reported information on weight and height. With regard to history of infertility, we asked whether there had been failure to achieve pregnancy after 24 months or more of regular unprotected intercourse.

For the present validation study, we mailed a follow-up questionnaire in 2012 to 1025 women who had answered that they had endometriosis by medical history at a baseline survey, to verify self-reported endometriosis based on our previous study.^16^ In the questionnaire, we again asked if the women had ever had physician-diagnosed endometriosis. If the answer was yes, they were asked to report when the diagnosis had been made and how endometriosis was found. In addition, we asked about the site of endometriosis determined by imaging, whether the women had undergone a surgical procedure and its content, and duration of treatment. A letter of informed consent was enclosed.

This study was approved by the Ethics Committee of Gunma University, Japan.

### Data analysis

A total of 15 019 female nurses participated in the JNHS follow-up cohort. We excluded data for logistic regression analysis from 163 women who did not respond to the validation study questionnaire and 20 who did not give information on history of endometriosis in the baseline survey. Data for 862 women with self-reported endometriosis were used for the validation study. We used the Cochran-Armitage trend test to assess trends in the proportions across the categories. Multinomial logistic regression models were used to estimate multivariable-adjusted odds ratios (ORs) and 95% confidence intervals (CIs) for women with imaging-diagnosed endometriosis and women with imaging-diagnosed adenomyosis, compared to control women without endometriosis and adenomyosis. We included women in the group of imaging-diagnosed endometriosis when both endometriosis and adenomyosis were found by imaging and in the group of imaging-diagnosed adenomyosis when only adenomyosis was found by imaging, regardless of whether or not surgery had been performed. Age at the survey, age at menarche (≤11, 12, 13, or ≥14 years), length of menstrual cycle at 18–22 years of age (≤25, 26–31, 32–49, or ≥50 days), smoking status at 30 years of age (never or ever), and BMI at 18 years of age (<18.5, 18.5–22.4, or ≥22.5) were included in the model as covariates. Tests for linear trend involved ordering categories of covariates and treating the values as continuous.

We used a forward stepwise logistic regression to determine which variables were predictors for propensity of undergoing surgery, including age at diagnosis, age at menarche, length of menstrual cycle at 18–22 years of age, number of deliveries, smoking status at 30 years of age, smoking status at diagnosis, BMI at 18 years of age, and history of infertility. The *P* value for entry was 0.05, and the *P* value to remain in the model was 0.05.

Infertility, which may affect the decision to undergo surgery, was used for subgroup analysis. Multinomial logistic regression models were used to compare characteristics of risk factors between endometriosis with surgery and endometriosis without surgery in the subgroups of women with and without a history of infertility.

A *P* value <0.05 was considered statistically significant. All statistical analyses were carried out using SAS ver 9.4 (SAS Institute Inc., Cary, NC, USA).

## RESULTS

Of 1025 women who noted that they had a self-reported history of endometriosis, 862 responded to our survey, and 638 reported physician-diagnosed endometriosis, suggesting that the positive predictive value was 74.0% (638/862; 95% CI, 70.9%–76.9%). As can be seen in Figure, 330 women answered that the sites of endometriosis were diagnosed by imaging procedures, such as ultrasonography and MRI. Of the 330 women with imaging-diagnosed endometriosis, 210 had endometriosis confirmed by a surgical procedure (Group A) and 120 had endometriosis that was not confirmed by a surgical procedure (Group B). Of the 210 women with surgically confirmed endometriosis, 170 had ovarian endometriosis; the sites of endometriosis in the other 40 women were the peritoneum, rectum, bladder, lung, and umbilicus. In the 638 women with physician-diagnosed endometriosis, 264 reported that the site of endometriosis was the uterus, namely adenomyosis (Group C). In women who responded, the proportions of imaging-diagnosed endometriosis and surgically confirmed endometriosis were 38.3% (330/862; 95% CI, 35.0%–41.6%) and 24.4% (210/862; 95% CI, 21.5%–27.4%), respectively. The overall prevalence of self-reported endometriosis was 6.94% (1025/14 762; 95% CI, 6.54%–7.37%).

**Figure.**
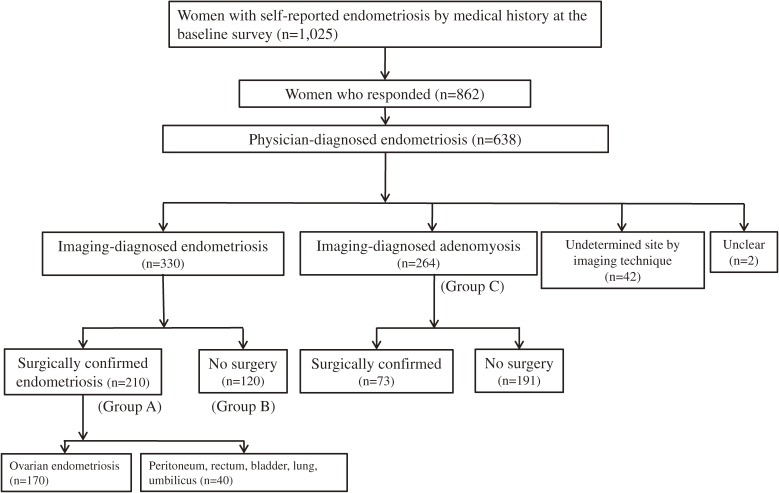
Decision tree for classification of women with self-reported endometriosis, physician-diagnosed endometriosis, imaging-diagnosed endometriosis, and surgically confirmed endometriosis.

### Comparison of risk profiles between imaging-diagnosed endometriosis and imaging-diagnosed adenomyosis

First, we compared risk profiles between imaging-diagnosed endometriosis (Group A plus Group B) and imaging-diagnosed adenomyosis (Group C). The proportion of women in Group C who were aged 45–54 years was higher than that in the imaging-diagnosed endometriosis group (Table 1). Mean ages at diagnosis were 32.0 years in the imaging-diagnosed endometriosis group and 33.9 years in the imaging-diagnosed adenomyosis group. In women with neither endometriosis nor adenomyosis, the proportion of women with a menstrual cycle ranging between 32 and 49 days in length was relatively high (21.6%), and the proportion of women with a history of infertility was relatively low (12.4%). The proportion of women who smoked cigarettes at 30 years of age was 20.0% in women with neither endometriosis nor adenomyosis. The proportions of women currently using oral contraceptives were low (1.1%–1.8%) in all groups. The proportions of women who were diagnosed in 1990–1999 were 47.0% in the imaging-diagnosed endometriosis group and 49.6% in the imaging-diagnosed adenomyosis group. As shown in Table 2, older age was associated with increased risk of adenomyosis. A short menstrual cycle at 18–22 years of age and cigarette smoking at 30 years of age were independently associated with risk of endometriosis (Group A plus Group B). In addition, women with low BMI at 18 years of age tended to be at high risk of adenomyosis, and those with older age at menarche tended to be associated with low risk of endometriosis.

**Table 1.** Baseline lifestyle and reproductive characteristics among women with imaging-diagnosed endometriosis, those with imaging-diagnosed adenomyosis, and those without either condition

		Imaging-diagnosed endometriosis		Imaging-diagnosed adenomyosis		Without endometriosis and adenomyosis	
		
		Number	Proportion	Number	Proportion	Number	Proportion
Age (years)	<35	76	23.0	29	11.0	3149	22.7
	35–44	150	45.5	114	43.2	5619	40.4
	45–54	89	27.0	98	37.1	4249	30.6
	55–64	14	4.2	21	8.0	833	6.0
	≥65	1	0.3	2	0.8	38	0.3
	Unknown	0	0.0	0	0.0	12	0.1

Age at diagnosis (years)	<30	132	40.0	88	33.3		
	30–39	141	42.7	97	36.7		
	≥40	50	15.2	74	28.0		
	Unknown	7	2.1	5	1.9		

Calendar year at diagnosis	<1990	91	27.6	84	31.8		
	1990–1999	155	47.0	131	49.6		
	≥2000	77	23.3	44	16.7		
	Unknown	7	2.1	5	1.9		

Age at menarche (years)	≤11	83	25.2	62	23.5	3086	22.2
	12	114	34.5	71	26.9	3997	28.8
	13	74	22.4	64	24.2	3234	23.3
	≥14	57	17.3	65	24.6	3484	25.1
	Unknown	2	0.6	2	0.8	99	0.7

Length of menstrual cycle at 18–22 years of age (days)	≤25	39	11.8	26	9.9	1232	8.9
	26–31	223	67.6	161	61.0	8213	59.1
	32–49	46	13.9	46	17.4	3007	21.6
	≥50	18	5.5	27	10.2	1242	8.9
	Unknown	4	1.2	4	1.5	206	1.5

Deliveries (numbers)	0	147	44.5	90	34.1	5218	37.5
	1	69	20.9	60	22.7	2773	20.0
	≥2	98	29.7	107	40.5	5437	39.1
	Unknown	16	4.8	7	2.7	472	3.4

Smoking at 30 years of age	Never	222	67.3	180	68.2	10 023	72.1
	Current smoker	80	24.2	64	24.2	2785	20.0
	Ex-smoker	26	7.9	18	6.8	986	7.1
	Unknown	2	0.6	2	0.8	106	0.8

BMI at 18 years of age	<18.5	31	9.4	42	15.9	1582	11.4
	≥18.5 and <22.5	213	64.5	154	58.3	8524	61.3
	≥22.5	71	21.5	58	22.0	3225	23.2
	Unknown	15	4.5	10	3.8	569	4.1

History of infertility	No	191	57.9	171	64.8	11 382	81.9
	Yes	127	38.5	79	29.9	1720	12.4
	Unknown	12	3.6	14	5.3	798	5.7

Current use of oral contraceptive for contraception	No	324	98.2	259	98.1	13 746	98.9
	Yes	6	1.8	5	1.9	154	1.1
	Unknown	0	0.0	0	0.0	0	0.0

BMI, body mass index.

**Table 2.** Reproductive and lifestyle factors and the risks of endometriosis and adenomyosis

		Imaging-diagnosed endometriosis				Imaging-diagnosed adenomyosis				Wald test between endometriosis and adenomyosis
	
		*n*	Cochran- Armitage trend test	Odds ratio	95% CI	*n*	Cochran- Armitage trend test	Odds ratio	95% CI	
Age (years)				0.991	0.977–1.01			1.04	1.02–1.05	χ^2^ = 20.4 *P* < 0.001

Age at menarche (years)	≤11	77	0.001	0.905	0.670–1.22	57	0.863	1.17	0.816–1.67	χ^2^ = 10.8 *P* = 0.095
	12	107		ref.		70		ref.		
	13	71		0.843	0.621–1.14	61		1.01	0.715–1.43	
	≥14	54		0.609	0.436–0.851	60		0.870	0.611–1.24	

Length of menstrual cycle at 18–22 years of age (days)	≤25	37	<0.001	1.19	0.837–1.70	25	0.579	1.07	0.698–1.64	χ^2^ = 20.0 *P* = 0.003
	26–31	211		ref.		153		ref.		
	32–49	43		0.557	0.400–0.776	46		0.856	0.613–1.20	
	≥50	18		0.569	0.349–0.928	24		1.16	0.748–1.80	

Smoking at 30 years of age	Never	207	0.046	ref.		169	0.155	ref.		χ^2^ = 8.2 *P* = 0.017
	Ever	102		1.31	1.03–1.67	79		1.30	0.990–1.71	

BMI at 18 years of age	<18.5	30	0.911	0.804	0.546–1.19	41	0.106	1.50	1.05–2.12	χ^2^ = 8.6 *P* = 0.072
	≥18.5 and <22.5	209		ref.		152		ref.		
	≥22.5	70		0.857	0.650–1.13	55		0.902	0.659–1.23	

BMI, body mass index; CI, confidence interval.

### Comparison of risk profiles between surgically confirmed endometriosis and imaging-diagnosed endometriosis without surgery

Baseline lifestyle and reproductive characteristics in Group A and Group B are shown in Table 3. By using the propensity score model, the tendency to have undergone no surgical procedure in Group A and Group B was significantly associated with multiparity (Wald test: *P* < 0.001) and cigarette smoking at 30 years of age (Wald test: *P* = 0.011). Women with a short menstrual cycle at 18–22 years of age had a significant risk of subsequently developing endometriosis in both Group A and Group B (test for linear trend in logistic regression: *P* < 0.001 and *P* = 0.017, respectively). This trend for risk of endometriosis was significantly different between the two groups (*P* = 0.005) (Table 4). There was a significant trend toward increased risk of endometriosis with younger age at menarche in Group A (test for linear trend in logistic regression: *P* < 0.001). Cigarette smoking at 30 years of age was associated with risk of endometriosis in Group B (test for linear trend in logistic regression: *P* = 0.008).

**Table 3.** Baseline lifestyle and reproductive characteristics between women with surgically confirmed endometriosis and those with imaging-diagnosed endometriosis without surgery

		Surgically confirmed endometriosis (Group A)		Imaging-diagnosed endometriosis without surgery (Group B)	
	
		Number	Proportion	Number	Proportion
Age (years)	<35	45	21.4	31	25.8
	35–44	93	44.3	57	47.5
	45–54	60	28.6	29	24.2
	55–64	11	5.2	3	2.5
	≥65	1	0.5	0	0.0
	Unknown	0	0.0	0	0.0

Age at diagnosis (years)	<30	76	36.2	56	46.7
	30–39	92	43.8	49	40.8
	≥40	36	17.1	14	11.7
	Unknown	6	2.9	1	0.8

Calendar year at diagnosis	<1990	56	26.7	35	29.2
	1990–1999	101	48.1	54	45.0
	≥2000	47	22.4	30	25.0
	Unknown	6	2.9	1	0.83

Age at menarche (years)	≤11	51	24.3	32	26.7
	12	79	37.6	35	29.2
	13	46	21.9	28	23.3
	≥14	33	15.7	24	20.0
	Unknown	1	0.5	1	0.8

Length of menstrual cycle at 18–22 years of age (days)	≤25	24	11.4	15	12.5
	26–31	144	68.6	79	65.8
	32–49	30	14.3	16	13.3
	≥50	10	4.8	8	6.7
	Unknown	2	1.0	2	1.7

Deliveries (numbers)	0	105	50.0	42	35.0
	1	46	21.9	23	19.2
	≥2	49	23.3	49	40.8
	Unknown	10	4.8	6	5.0

Smoking at 30 years of age	Never	148	70.5	74	61.7
	Current smoker	45	21.4	35	29.1
	Ex-smoker	15	7.1	11	9.2
	Unknown	2	1.0	0	0.0

BMI at 18 years of age	<18.5	17	8.1	14	11.7
	≥18.5 and <22.5	138	65.7	75	62.5
	≥22.5	44	21.0	27	22.5
	Unknown	11	5.2	4	3.3

History of infertility	No	109	51.9	82	68.3
	Yes	93	44.3	34	28.3
	Unknown	8	3.8	4	3.3

Current use of oral contraceptive for contraception	No	208	99.0	116	96.7
	Yes	2	1.0	4	3.3
	Unknown	0	0.0	0	0.0

BMI, body mass index.

**Table 4.** Reproductive and lifestyle factors versus risk of endometriosis

		Surgically confirmed endometriosis (Group A)				Imaging-diagnosed endometriosis without surgery (Group B)				Wald test between Group A and Group B
	
		*n*	Cochran- Armitage trend test	Odds ratio	95% CI	*n*	Cochran- Armitage trend test	Odds ratio	95% CI	
Age (years)				0.998	0.980–1.02			0.979	0.956–1.00	χ^2^ = 2.9 *P* = 0.238

Age at menarche (years)	≤11	47	0.003	0.811	0.559–1.18	30	0.148	1.12	0.677–1.85	χ^2^ = 11.4 *P* = 0.077
	12	74		ref.		33		ref.		
	13	43		0.730	0.499–1.07	28		1.10	0.661–1.83	
	≥14	31		0.504	0.329–0.771	23		0.850	0.495–1.46	

Length of menstrual cycle at 18–22 years of age (days)	≤25	23	<0.001	1.17	0.750–1.83	14	0.017	1.23	0.694–2.19	χ^2^ = 18.5 *P* = 0.005
	26–31	134		ref.		77		ref.		
	32–49	28		0.580	0.385–0.875	15		0.520	0.298–0.906	
	≥50	10		0.518	0.271–0.992	8		0.649	0.311–1.36	

Smoking at 30 years of age	Never	137	0.628	ref.		70	0.007	ref.		χ^2^ = 7.1 *P* = 0.028
	Ever	58		1.14	0.834–1.55	44		1.64	1.12–2.40	

BMI at 18 years of age	<18.5	16	0.767	0.674	0.399–1.14	14	0.838	1.03	0.579–1.84	χ^2^ = 3.1 *P* = 0.538
	≥18.5 and <22.5	135		ref.		74		ref.		
	≥22.5	44		0.832	0.589–1.18	26		0.898	0.571–1.41	

BMI, body mass index; CI, confidence interval.

### Comparison of risk profiles for women with and without a history of infertility

We examined risk factors in two stratified population—women with and without a history of infertility (*n* = 1993 and *n* = 11 934, respectively)—because infertility may be involved in the decision to perform surgery. In women with a history of infertility, a short menstrual cycle at 18–22 years of age was associated with significantly increased risk of endometriosis in both Group A and Group B (test for linear trend in logistic regression: *P* = 0.001 and *P* = 0.005, respectively). This trend for risk of endometriosis was significantly different between the two groups (*P* = 0.006) (Table 5). There was a significant trend toward increased risk of endometriosis with younger age at menarche in Group A (test for linear trend in logistic regression: *P* = 0.037).

**Table 5.** Reproductive and lifestyle factors versus risk of endometriosis in women with a history of infertility

		Surgically confirmed endometriosis (Group A)				Imaging-diagnosed endometriosis without surgery (Group B)				Wald test between Group A and Group B
	
		*n*	Cochran- Armitage trend test	Odds ratio	95% CI	*n*	Cochran- Armitage trend test	Odds ratio	95% CI	
Age (years)				0.995	0.964–1.03			0.959	0.908–1.01	χ^2^ = 2.4 *P* = 0.308

Age at menarche (years)	≤11	22	0.016	0.771	0.435–1.37	10	0.081	0.934	0.386–2.26	χ^2^ = 3.2 *P* = 0.786
	12	31		ref.		11		ref.		
	13	18		0.751	0.411–1.37	5		0.630	0.215–1.85	
	≥14	14		0.614	0.319–1.18	6		0.767	0.276–2.13	

Length of menstrual cycle at 18–22 years of age (days)	≤25	12	0.005	1.30	0.680–2.50	6	0.011	1.68	0.667–4.24	χ^2^ = 18.0 *P* = 0.006
	26–31	59		ref.		23		ref.		
	32–49	10		0.430	0.217–0.851	1		0.106	0.014–0.792	
	≥50	4		0.369	0.132–1.04	2		0.427	0.099–1.85	

Smoking at 30 years of age	Never	60	0.666	ref.		21	0.299	ref.		χ^2^ = 0.3 *P* = 0.850
	Ever	25		1.05	0.645–1.71	11		1.23	0.582–2.60	

BMI at 18 years of age	<18.5	5	0.196	0.424	0.167–1.08	4	0.598	1.02	0.340–3.05	χ^2^ = 4.2 *P* = 0.384
	≥18.5 and <22.5	56		ref.		19		ref.		
	≥22.5	24		1.15	0.692–1.90	9		1.24	0.545–2.82	

BMI, body mass index; CI, confidence interval.

In women without a history of infertility, a short menstrual cycle at 18–22 years of age was associated with a significantly increased risk of endometriosis in Group A (test for linear trend in logistic regression: *P* = 0.045), but not in Group B. There was a significant trend toward increased risk of endometriosis with younger age at menarche in Group A (test for linear trend in logistic regression: *P* = 0.013). Cigarette smoking at 30 years of age was associated with risk of endometriosis in Group B (test for linear trend in logistic regression: *P* = 0.008). This trend for risk of endometriosis was significantly different between the two groups (*P* = 0.011) (Table 6).

**Table 6.** Reproductive and lifestyle factors versus risk of endometriosis in women without a history of infertility

		Surgically confirmed endometriosis (Group A)				Imaging-diagnosed endometriosis without surgery (Group B)				Wald test between Group A and Group B
	
		*n*	Cochran- Armitage trend test	Odds ratio	95% CI	*n*	Cochran- Armitage trend test	Odds ratio	95% CI	
Age (years)				1.00	0.975–1.03			0.987	0.959–1.02	χ^2^ = 0.8 *P* = 0.670

Age at menarche (years)	≤11	25	0.053	0.817	0.492–1.36	20	0.406	1.26	0.673–2.36	χ^2^ = 9.6 *P* = 0.144
	12	40		ref.		20		ref.		
	13	24		0.740	0.444–1.23	23		1.46	0.797–2.67	
	≥14	15		0.441	0.242–0.805	16		0.957	0.492–1.86	

Length of menstrual cycle at 18–22 years of age (days)	≤25	10	0.045	0.987	0.507–1.92	8	0.217	1.06	0.502–2.24	χ^2^ = 5.4 *P* = 0.500
	26–31	71		ref.		52		ref.		
	32–49	17		0.665	0.390–1.13	13		0.665	0.361–1.23	
	≥50	6		0.605	0.260–1.40	6		0.751	0.319–1.77	

Smoking at 30 years of age	Never	73	0.797	ref.		46	0.009	ref.		χ^2^ = 9.1 *P* = 0.011
	Ever	31		1.19	0.780–1.82	33		1.96	1.25–3.07	

BMI at 18 years of age	<18.5	10	0.525	0.794	0.408–1.54	9	0.740	0.945	0.464–1.93	χ^2^ = 3.6 *P* = 0.462
	≥18.5 and <22.5	75		ref.		53		ref.		
	≥22.5	19		0.641	0.386–1.07	17		0.818	0.471–1.42	

BMI, body mass index; CI, confidence interval.

## DISCUSSION

In a validation study of self-reported endometriosis, diagnosis of endometriosis by laparoscopy was confirmed in 89% of randomly selected women.^17^ In the present study, the proportion of women with surgically confirmed endometriosis in women with physician-diagnosed endometriosis was 32.9% (95% CI, 29.3–36.7%), but the proportion in women with imaging-diagnosed endometriosis excluding adenomyosis was 51.7% (95% CI, 47.8%–55.7%). In the past in Japan, adenomyosis has been included in endometriosis in a broad sense. Therefore, exclusion of adenomyosis by imaging techniques is needed to assess the predictive value of endometriosis in an epidemiological survey. It has been reported that noninvasive procedures such as ultrasound sonography have had moderate success in predicting a surgical diagnosis of endometriosis.^9^ Imaging techniques may play an important role in the prediction of surgically diagnosed endometriosis. Thus, the results of the present prospective study in women with imaging-diagnosed endometriosis may be reliable.

In case-control studies, selection of appropriate controls is difficult because of the requirement of a surgical diagnosis. Fertile women who underwent laparoscopic sterilization and infertile women who underwent laparoscopy for diagnosis and treatment unrelated to endometriosis have sometimes been included in control groups of such studies.^7^ However, it has been demonstrated that these women are unlikely to be representative of the symptomatic population from which cases were drawn.^18^ Missmer et al suggested that this procedure resulted in overmatching and attenuation of relative risks for some exposures, since the controls consisted of women who had received surgical pelvic investigation for other reasons, such as tubal ligation.^17^ In addition, women with severe endometriosis are likely to receive a surgical procedure. On the other hand, most community- and hospital-based controls did not have endometriosis ruled out by laparoscopy, raising the possibility of disease misclassification.^18^ Zondervan reported that the community prevalence of advanced stages of endometriosis is probably less than 2%, suggesting that community-based control groups are unlikely to include many undiagnosed cases if they are screened for moderate to severe pelvic symptoms.^7^ Based on our results, an imaging technique without a surgical procedure may be a suitable predictor of surgically confirmed endometriosis, suggesting that imaging techniques are useful for an epidemiological survey of endometriosis in both population-based and case-control studies. In addition, imaging-diagnosed endometriosis may be useful for assessment of the onset of risk of endometriosis-related diseases in the future.

Several risk factors, including lifestyle, environmental, sociodemographic, reproductive, and genetic characteristics, have been shown to be associated with endometriosis.^19^ It has been reported that a short menstrual cycle is associated with risk of endometriosis^20^^,^^21^ and that women with irregular menstrual cycles have a lower risk of endometriosis than that with regular menstrual cycle.^22^ Missmer et al reported a modest effect of menstrual cycle length on the risk of endometriosis.^4^ In the present study, a short menstrual cycle was associated with risk of endometriosis in both women with surgically confirmed endometriosis and women with imaging-diagnosed endometriosis without surgery. In addition, multiparous women and women with habitual cigarette smoking were less likely to undergo surgery. Since the low number of deliveries indicates a result caused by endometriosis, and since smoking cessation is expected to be more common among surgery patients, surgically confirmed endometriosis and imaging-diagnosed endometriosis without surgery have a similar risk profiles. Cigarette smoking at 30 years of age might be considered as having a status of “smoking” prior to diagnosis, since mean ages at diagnosis were 32.0 and 33.9 years in the imaging-diagnosed endometriosis and imaging-diagnosed adenomyosis groups, respectively. Although the proportions of women whose age at diagnosis was less than 30 years were 33%–40%, it is possible but unlikely that endometriosis was the reason for starting to smoke. Quite the contrary, cigarette smoking is considered to be a risk factor for occurrence of endometriosis. The prevalence of cigarette smoking at 30 years of age in women without endometriosis and adenomyosis (20.0%) was slightly higher than that in women of 30–39 years of age in the Japanese general female population (18.1%).^23^ However, we have no reason to suspect that the general population of women would differ in terms of the association observed in this study between cigarette smoking and endometriosis.

We examined whether risk factors of endometriosis differ according to the presence of a history of infertility. In previous studies, women with infertility were excluded or both fertile and infertile women were included in recruited subjects. To date, there have been few reports regarding the risk factors of endometriosis in women with a history of infertility. It has been reported that an increased risk of endometriosis among women with natural red hair was found in women with infertility.^14^ Recently, Peterson et al reported that a history of infertility was a risk factor for endometriosis in both a surgically diagnosed cohort and an imaging-diagnosed cohort.^13^ In women with a history of infertility, we found that shorter menstruation was associated with an increase in the risk of endometriosis in both women with surgically confirmed endometriosis and women with imaging-diagnosed endometriosis without surgery. On the other hand, risk factors for imaging-diagnosed endometriosis without surgery were different between women with and those without a history of infertility, although risk factors for surgically confirmed endometriosis were similar, regardless of a history of infertility.

Results of studies on the effect of smoking on endometriosis have been conflicting.^17^^,^^19^ Chapron et al reported that smoking was not an independent risk factor for endometriosis in a population of women with histologically severe endometriosis.^19^ Missmer et al reported that current smoking was associated with reduced risk for endometriosis.^17^ In the present study, women with no history of infertility who smoked cigarettes at the age of 30 years were unlikely to request a surgical procedure for endometriosis. We speculate that imaging-diagnosed endometriosis without surgery and surgically confirmed endometriosis can be considered to be the same entity, but indication for and willingness to undergo surgery in women without a history of infertility were different from those in women with a history of infertility, because the background characteristics were different. Based on the results, the presence of a history of infertility should be taken into consideration when conducting an epidemiological survey of endometriosis.

Adenomyosis, which is defined as the presence of endometrial glands and stroma deep within the myometrium, is identified as an entity separate from endometriosis. Therefore, risk factors for adenomyosis should be discriminated from risk factors for endometriosis. Yeniel et al reported that the rates of smoking, previous uterine surgery, and nulliparity in women with adenomyosis were significantly higher than those in women without adenomyosis with respect to epidemiological, clinical, and histopathological characteristics.^24^ Shrestha reported that multiparity, smoking, and irregular menstruation cycle increased the risk of developing adenomyosis.^25^ We showed that risk profiles in women with adenomyosis diagnosed by imaging techniques were inconsistent with those in women with imaging-diagnosed endometriosis (Group A and Group B, respectively). Therefore, endometriosis and adenomyosis should be considered two different entities.

This study has notable strengths. The large sample size of the JNHS offers a unique opportunity to add to the limited knowledge of the validation of endometriosis diagnosis. We clarified that imaging-diagnosed endometriosis is a useful assessment method for an epidemiological study. In addition, differences in the risk factors for endometriosis between women with and without a history of infertility were demonstrated.

Some methodological limitations should be addressed. Retrospective recall by individual women, such as recall of the menstrual cycle, smoking habit, body weight and height, is a limitation of this study. Information about endometriosis reported by nurses would be more accurate than that reported by the general population, since nurses should have a better understanding of questions about endometriosis. Also, the ability to determine causation is limited in this study due to its cross-sectional nature. We did not include educational attainment and other socioeconomic indicators as risk factors, since the population in this study was composed of women with similar educational and socioeconomic backgrounds. In addition, completed questionnaires represent only 45.4% of 110 000 copies of the baseline questionnaire, which accounted for 8.6% of the members of the Japan Nursing Association (in 2005) or 0.104% of the female general population aged over 25 years in Japan.^15^ Therefore, the results may not be representative of the entire Japanese female population. Assessment of the risk for occurrence of endometriosis-related diseases in the future may be necessary in the population of women with imaging-diagnosed endometriosis. A prospective study on the differences in risk profiles between women with surgically confirmed endometriosis and women with imaging-diagnosed endometriosis without surgery is also needed. In the present study, year at diagnosis ranged over a period of many years. Improvements in imaging technology might affect the accuracy of imaging-based diagnosis, even though previous diagnoses have also been made using ultrasound sonography. Finally, our study suggests that the validity of imaging-diagnosed endometriosis without surgery may be appropriate for moderate or severe endometriosis but not minimal or mild endometriosis. Further study on the accuracy of imaging-based diagnosis of different degrees of severity of endometriosis is needed.

### Conclusions

Women with surgically confirmed endometriosis and those with imaging-diagnosed endometriosis without surgery have basically common risk profiles and may be used as the same disease entity for an epidemiological survey. However, in women without a history of infertility, it is necessary to pay attention to the differences in indication and preference for surgery according to background characteristics.

## ONLINE ONLY MATERIAL

Abstract in Japanese.
