# Supplementary material for: Risk Profiles for Endometriosis in Japanese Women: Results From a Repeated Survey of Self-Reports
Source: J Epidemiol. 2015 Mar 5;25(3):194–203. doi: 10.2188/jea.JE20140124 (PMC4340996; doi:10.2188/jea.JE20140124)
Supplement: Abstract in Japanese. [file je-25-194-s001.pdf]

本邦女性における子宮内膜症の危険因子

安井敏之[1)]、林邦彦[2)]、長井万恵[2)]、水沼英樹[3)]、久保田俊郎[4)]、李廷秀[5)]、鈴木庄亮[6)]

1) 徳島大学大学院ヘルスバイオサイエンス研究部生殖補助医療学分野
2) 群馬大学大学院保健学研究科
3) 弘前大学医学部産科婦人科
4) 東京医科歯科大学大学院医歯学総合研究科生殖機能協関学
5) 東京大学大学院医学系研究科公共健康医学専攻健康増進科学分野
6) 群馬大学名誉教授

Key words：子宮内膜症、危険因子、不妊症、手術、画像

背景： 子宮内膜症の発症や危険因子に関する研究結果は、対象者の選択や子宮内膜症の診断方法によって異なる可能性が考えられる。私たちは本邦女性において画像検査によって診断された子宮内膜症と手術によって診断された子宮内膜症について、リスク因子が異なるのかどうかを検討し、同時に子宮内膜症に影響を及ぼす不妊症の既往の有無によってそれらのリスク因子に違いが存在するかどうかを検討した。

方法： 日本ナースヘルス研究のベースライン調査（14945 名）において子宮内膜症と回答した 1025 名に対して、画像検査や手術に関する内容や部位を含めた子宮内膜症に関する詳細な質問票を送り、回答してもらった。

結果： 手術により子宮内膜症と診断された群（A 群）は 210 名、手術は行わず画像検査のみで子宮内膜症と診断された群（B 群）は 120 名、子宮腺筋症と診断された群（C 群）は 264 名であった。子宮内膜症と診断された A 群＋B 群におけるリスク因子は、18〜22 歳時における短い月経周期、30 歳時における喫煙習慣であったが、子宮腺筋症である C 群では年齢がリスク因子となっており、両疾患におけるリスク因子は異なっていた。また、不妊症の既往がある女性では、A 群および B 群いずれも、18〜22 歳時における短い月経周期がリスク因子であったが、不妊症の既往がない女性では A 群および B 群とでリスク因子が異なっていた。

結論： 手術によって子宮内膜症と診断された群と画像検査のみで子宮内膜症と診断された群はいずれも基本的には共通のリスク因子を有しており、子宮腺筋症のリスク因子とは異なっていた。また、子宮内膜症のリスク因子を検討するためには不妊症の既往の有無を考慮すべきである。
